# Effectiveness of the bucco-lingual technique within a school-based supervised toothbrushing program on preventing caries: a randomized controlled trial

**DOI:** 10.1186/1472-6831-11-11

**Published:** 2011-03-22

**Authors:** Paulo Frazão

**Affiliations:** 1 Public Health School, University of São Paulo, São Paulo, Brazil

## Abstract

**Background:**

Supervised toothbrushing programs using fluoride dentifrice have reduced caries increment. However there is no information about the effectiveness of the professional cross-brushing technique within a community intervention. The aim was to assess if the bucco-lingual technique can increase the effectiveness of a school-based supervised toothbrushing program on preventing caries.

**Methods:**

A randomized double-blinded controlled community intervention trial to be analyzed at an individual level was conducted in a Brazilian low-income fluoridated area. Six preschools were randomly assigned to the test and control groups and 284 five-year-old children presenting at least one permanent molar with emerged/sound occlusal surface participated. In control group, oral health education and dental plaque dying followed by toothbrushing with fluoride dentifrice supervised directly by a dental assistant, was developed four times per year. At the remaining school days the children brushed their teeth under indirect supervising of the teachers. In test group, children also underwent a professional cross-brushing on surfaces of first permanent molar rendered by a specially trained dental assistant five times per year. Enamel and dentin caries were recorded on buccal, occlusal and lingual surfaces of permanent molars during 18-month follow-up. Exposure time of surfaces was calculated and incidence density ratio was estimated using Poisson regression model.

**Results:**

Difference of 21.6 lesions per 1,000 children between control and test groups was observed. Among boys whose caries risk was higher compared to girls, incidence density was 50% lower in test group (p = 0.016).

**Conclusion:**

Modified program was effective among the boys. It is licit to project a relevant effect in a larger period suggesting in a broader population substantial reduction of dental care needs.

**Trial registration:**

ISRCTN18548869.

## Background

Despite its decreasing prevalence in schoolchildren, dental caries is still an important public health problem [1]. The occlusal fissured surface of the first permanent molars and their lower buccal and upper lingual pits are among the most susceptible sites for caries [2].

Sound surfaces are more protected by fluoride treatments than deep pits and fissures of occlusal surfaces. The sealant of pits and fissures is a recommended procedure to prevent occlusal caries of permanent molars. Its effectiveness is clear at high caries risk but information on the benefits of sealing specific to different caries risks is lacking [3]. Moreover the cost-effectiveness of its application can be high if factors as durability, tooth selection, expenditures, operator technique, monitoring and combined use with other preventive measures are not controlled [4].

As an alternative technique, researchers showed that a non-operative treatment program designed to control occlusal caries on the basis of intensive patient education and professional toothcleaning could maintain the integrity of occlusal surface without systematic use of sealants [5]. Recent studies have demonstrated the efficacy of plaque removal by cross-brushing technique on partially erupted occlusal surfaces [6,7] however there is no information about its effectiveness within a community intervention. In addition, supervised toothbrushing programs using fluoride dentifrice have reduced caries increment [8,9]. Such programs are a basic public health strategy in the dental caries prevention widely recommended for assuring affordable fluoride dentifrice [10]. Toothbrushes are also provided, but it is more important to maintain periodical activities for reinforcing their optimal use than simply to distribute them.

The planning and focusing of preventive measures is more difficult in low caries prevalence population reason why such activities must be avoided before their effectiveness have been demonstrated and the individuals under risk have been evaluated [11]. A basic preventive program can be more cost-effective than an intensive program for risk individuals [12]. In the majority of the contexts and even more when available resources are limited, overtreatment must be avoided. In low prevalence levels, the majority of the lesions reaches a minority of the individuals however the development of new lesions is not restricted to individuals in the risk group and occurs in the population as a whole. This characteristic requires population-based strategy combined to the risk strategy [13].

Although programs based on strategies related to individualized needs tend to be more effective [14,15] they require more specialized personnel reason why they can be more expensive compared to population-based strategy programs. The cost-effectiveness can not be justified faced on the programs which auxiliary personnel can be employed and simpler measures can be planned to identify risk conditions and to detect new lesions as well as to undertake health promotion and disease prevention actions. Evidence related to professional cross-brushing on erupting permanent molars within community interventions such as school-based supervised toothbrushing programs still was not explored.

The purpose of this study was to assess if the bucco-lingual technique provided by a trained dental assistant can increase the effectiveness of a school-based supervised toothbrushing program on preventing caries.

## Methods

### Study population

An 18-month randomized double-blinded controlled community intervention trial to be analyzed at an individual level was conducted from May, 2007, to November, 2008, in a dynamic cohort of preschool children from six units located in a low-income fluoridated (0.7 mgF/L) area within the city of Sao Vicente, Brazil.

Brazilian schoolchildren have experienced caries decline in the last years [16]. Sao Vicente and several cities have undertaken school-based supervised toothbrushing programs financed by the Brazilian Health System [17]. Many dental assistants and a great amount of material resources are spent yearly. This action comprehends oral health education and dental plaque dying followed by supervised toothbrushing with fluoride dentifrice carried out quarterly by dental auxiliary personnel [18]. Oral health care has been postulated as one of the strategies toward primary health care (PHC) and the coverage of the abovementioned action has been an indicator among others assumed in the PHC statement.

The selected preschools were located in the same region within the peripheral insular area of the Sao Vicente and shared similar curriculum framework and socio-environmental context. The preschools were randomly assigned to the test and control groups. All five-year-old schoolchildren presenting at least one permanent molar with one or more emerged and sound surfaces were considered eligible.

### Interventions

The conventional program, composed by oral health education and dental plaque dying followed by toothbrushing with fluoride dentifrice (1,100 μgF/g) supervised directly by a dental assistant, was developed four times per year in the control units. The dental assistant was not skilled on special toothbrushing methods for erupting molars and was not trained to carry out the cross-brushing technique. At the remaining school days the children brushed their teeth under indirect supervising of the teachers. The toothbrushing was carried out in the indoor recreation area of each preschool. Beyond this conventional basic activity, children underwent professional cross-brushing on surfaces of first permanent molar, rendered by a specially trained dental assistant, five times per year in the test units. The supervised toothbrushing program has begun from the second month of each year and has finished before the last month because the preschools do not have had a regular functioning on January and December. About 14 days were spent in each time for both. In other terms, the modified program was applied every about 45 days from February to November while the conventional one was carried out every about 60 days in the same period.

One of the reasons for the high caries prevalence in occlusal surfaces of first permanent molars is their relatively long eruption phase, during which they are not cleaned with normal horizontal toothbrushing. Based on this fact, professional cross-brushing for erupting molars has been proposed. The toothbrush must be held in the bucco-lingual direction with the bristles towards the occlusal surface and moved with small rotary motions [5]. The interventions were controlled by periodical checking for supervising the activities.

### Oral examinations

One calibrated dentist was responsible for all of the dental examinations. Before the first and the third exam, the intra-observer and inter-observer agreement with a gold-standard public health dental practitioner (MHMG) were both checked. The participants were kept unaware if they belonged to control or test units. The examiner was kept masked to group assignment and the dental assistant in charge of the control units was kept blinded about differential characteristics of the intervention in the test preschools.

The children did not brush their teeth under supervision of the dental assistants prior to the examinations that were carried out at preschool using the WHO probe and a flat mirror at different period from that employed for interventions rendered by dental assistants. Birth date and gender of the subjects were obtained. The participants were classified according to skin color as this characteristic can be associated with dental caries [19]. Data for summarizing decayed, missing and filling deciduous teeth (dmft) were gathered at baseline. Only those deciduous teeth extracted for caries were considered missing [20]. The area behind second deciduous molars was examined for all observations. The space was classified according to the emergence of the first permanent molar. The surfaces of emerged permanent molars were categorized as healthy - no evidence of cavity or tissue loss equal or higher than 0.5 mm; as enamel caries - with at least 0.5 mm of tissue loss and no evidence that cavitation has penetrated the dentin [21]; as dentin caries - tissue loss reaching dentin or presence of unmistakable cavity, undermined enamel or a detectably softened floor or wall [20]; and as filled - presence of sealant or definitive/temporary filling. The probe was used to clean the tooth surface from plaque and debris prior to examination and to check the surface texture of a lesion without penetrating it. Fluoride levels in drinking water and in dentifrice were controlled during the trial.

### Ethical approval and parental consent

The protocol was approved by the Ethics Committee from Catholic University of Santos (4843-20-2005). The parents of all participants gave signed informed consent. The study was registered in the Current Controlled Trials website http://www.controlled-trials.com sponsored by BioMed Central Ltd under the number ISRCTN 18548869.

### Data analysis

Although the study has used the preschool as the unit of randomization in order to avoid contamination, the individual level was the primary focus of the study outcome and was adopted as the inference level. Sample size estimation with a 95% confidence interval and statistical power of 80% showed that 247 children with at least six emerged surfaces at risk permitted to assess a ratio ≥ 2 between two groups considering an estimate of 2.5% (3 decayed surfaces in each 120 surfaces) in the control group [22]. Intra-observer and inter-observer agreements were measured with the Kappa test.

The participation of the children was controlled. Those who had five or more absences per month in preschool were excluded of the analysis.

Chi-square, t-Student and Mann-Whitney tests were used in order to identify differences in the baseline characteristics of test and control groups. The number of exposed surfaces and the exposure time (in months) since the last exam were calculated in each follow-up. Beyond occlusal surfaces, the buccal and lingual ones considered as similar risk [2] were included. The product of these two measurements is the surfaces-month value. The incidence density corresponds to the number of surfaces presenting enamel/dentin lesions divided by the number of exposed surfaces-month. Incidence density rate and 95% confidence intervals were obtained as the main response variable.

The individual-level analysis was adopted in order to control differences related to exposed surfaces, age and dmft of each participant. A model adjusted for age and caries-risk indicator was used to test for outcome difference between the modified and conventional program groups after 18-month follow-up. Multiple analyses were undertaken using Poisson regression model [23]. Incidence density ratio was estimated adopting p < 0.05 to reject null hypothesis. The analysis was stratified by gender due to the dmft difference identified at baseline. The Stata program (version 10.0) was used.

## Results

Kappa values for intra-observer and inter-observer agreements were excellent (>0.93). Fluoride levels in samples of drinking water and utilized dentifrice presented respectively 0.7 mgF/L and 1,100 μgF/g.

**Figure 1:**
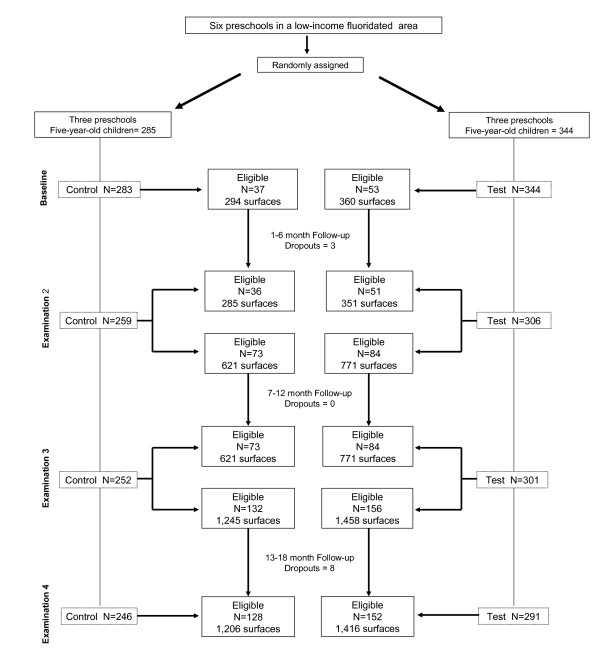
Flow of the participants through each stage

Figure 1 shows the participants flow through each stage. The lateral columns describe the number of the examined participants and the central columns the eligible participants in each turn. At baseline 283 subjects in the control preschools and 344 in the test units were examined. Out of 37 and 53 eligible participants, 36 and 51 were reexamined after six months as showed in the central columns. Out of 73 and 84 eligible participants in the second examination all were reexamined after six months and so on. The loss of eligible participants was noted in the first and the third follow-ups. The rates were 3.3% and 2.7% respectively due to participants' absences in preschool. No difference was observed between gender participation in control and test groups.

**Figure 2:**
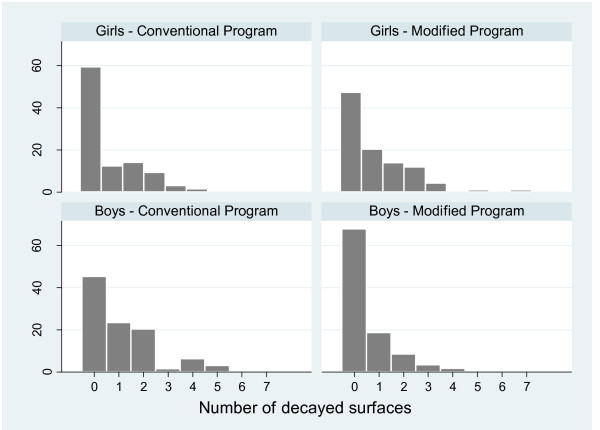
**Relative frequency of enamel and dentin caries incidence values distribution according to gender in the conventional^† ^and modified^†† ^program**. ^† ^Oral health education + daily toothbrushing with fluoride dentifrice + supervised toothbrushing program with fluoride dentifrice (1,100 μgF/g) four times per year; ^†† ^Conventional + professional cross-brushing five times per year.

Enamel/dentin caries was analyzed in 284 children that fulfilled eligibility criterion. The study population characteristics in control and test groups are illustrated in Table 1. No statistically significant differences were observed according to gender, skin color, age and dmft. Histograms show the distribution of enamel and dentin caries incidence values for the girls and boys in accordance with the program (Figure 2). Out of the participants, 130 children had one follow-up, 68 had two and 86 had three follow-ups comprising 524 measurements.

**Table 1:** Study population characteristics in the control and test units according to gender, skin color, age and dmft

		Control	Test	P value
Variables	Categories	n (%)	n (%)	
Gender	Female	66 ( 50.8)	94 ( 61.0)	
	Male	64 ( 49.2)	60 ( 39.0)	0.082^††^
Skin color	Mixed	117 ( 90.0)	139 ( 90.4)	
	Others^†^	13 ( 10.0)	15 ( 9.6)	0.899^††^

TOTAL		130 (100.0)	154 (100.0)	

		Mean (SD) Median	Mean (SD) Median	

Age (months)		68.40 (4.67) 68.57	68.56 (4.93) 68.73	0.786*
Dmft		2.02 (2.99) 0.00	2.27 (3.41) 1.00	0.657**

São Vicente, SP, Brazil.

^† ^Whites+Blacks+Yellows; ^†† ^Pearson Chi-square; * Student Test "t"; ** Mann-Whitney Test

A difference of 1.8 lesions per 1,000 exposed surfaces-month between control and test groups during the 18-month follow-up was observed corresponding to 18 lesions in each 10,000 surfaces (Table 2). After the adjusting of the estimates for 1,000 children (12,000 surfaces) the difference yielded 21.6 lesions per 1,000 children between the groups. Disparity was noted between the values for girls and boys, respectively 14 and 63 lesions per 10,000 surfaces-month implying a difference around 16.8 and 75.6 lesions per 1,000 children. More favorable incidence density values were observed in the caries-free group at the follow-up beginning. According to the intervention, lower values were observed in the boys, reason why the dmft values were checked for each gender. The dmft mean values for the girls and the boys were respectively 1.75 (SD 2.65) and 2.69 (SD 3.79) with a p value equal to 0.05 by the Mann-Whitney test. There was no statistically significant difference for age mean between girls and boys. Then the multiple analysis results were presented for each gender (Table 3). The incidence rate was 10% higher in children presenting at least one decayed deciduous tooth (girls IDR = 1.11 95%CI 1.01-1.22 p = 0.026 and boys IDR = 1.10 95%CI 1.02-1.20 p = 0.014). The modified program produced incidence values statistically more favorable in the boys independently of the variation of their caries experience and their age. Incidence density was 50% lower in the test group compared to the control group (IDR = 0.48 95%CI 0.27-0.87 p = 0.016).

**Table 2:** Incidence density for caries* (per 1,000 exposed surfaces-month) according to gender and caries experience at deciduous dentition (dmft) in the control and test units

	Total	Control^†^	Test^††^
*Gender*			
Female	11.3 [8.7 - 13.7]	10.4 [6.7 - 14.1]	11.8 [8.4 - 15.3]
Male	9.0 [6.6 - 11.4]	12.0 [8.3 - 15.6]	**5.7 [3.0 - 8.4]**
*Dmft*			
= 0	**6.1 [4.1 - 8.1]**	**6.6 [4.0 - 9.3]**	**5.6 [2.8 - 8.4]**
> 0	14.4 [11.6 - 17.1]	16.1 [11.9 - 20.2]	13.0 [9.4 - 16.5]

Total	10.2 [8.5 - 12.0]	11.2 [8.6 - 13.8]	9.4 [7.0 - 11.7]

Confidence interval and point estimates. São Vicente, SP, Brazil.

* Enamel + dentin lesions; ^† ^Oral health education + daily toothbrushing with fluoride dentifrice + supervised toothbrushing program with fluoride dentifrice (1,100 μgF/g) four times per year;

^†† ^Conventional + professional cross-brushing five times per year.

**Table 3:** Incidence density ratio (IDR) for enamel and dentin caries according to age, caries experience at deciduous dentition (dmft) and the program for the girls and the boys

	Girls			Boys		
	
Variables	IDR	CI 95%	P*	IDR	CI 95%	P*
age (months)**	1.01	0.97-1.05	0.649	0.97	0.93-1.02	0.221
dmft^†^	1.11	1.01-1.23	0.026	1.10	1.02-1.20	0.014
Program^††^	1.34	0.77-2.34	0.294	0.48	0.27-0.87	0.016

São Vicente, SP, Brazil. Estimates by Poisson regression.

* Wald test ** At the follow-up beginning ^† ^dmft = 0 as reference category ^†† ^Conventional program as reference category

## Discussion

This trial aimed to test if the bucco-lingual technique could increase the effectiveness of a school-based supervised toothbrushing program on preventing caries. The modified program was effective among the boys independently of the variation of their caries experience and their age. Incidence rate was 50% lower in the test group compared to the control group. Differences in the incidence rates were observed among the caries-free participants of both genders compared to the others, corroborating to the evidences on the dmft as a risk indicator for future caries [24,25]. Among the girls no significant difference was observed. One of the reasons for this output can be related to the caries disparity observed at baseline between the genders. The modified program was effective in the higher-risk gender. The professional cross-brushing for erupting molars [5-7] and the lower timing interval introduced in the supervised toothbrushing program can explain the observed effect among the boys.

Because the interventional characteristics, the use of individual randomization was assumed as impractical. Children move among classes particularly from a year to another and to avoid possible interferences in the same unit, the randomization was based on the preschool. Because the study was not looking for cross-level interactions it did not have more than six units. The participation of several units from different areas in the city could imply significant context differences beyond several operational difficulties and budget constraints. The selected units were located in the same social privation area and shared the same fluoride levels in the drinking water reason why we assumed no exposure differences among the participants related to the characteristics of the selected preschools.

Although randomization by preschool has been adopted, the analysis was based on the individuals according to the gender and the program. The compared groups did not present significant differences in the age, skin color, gender composition and caries experience at baseline. Skin color variable may be a proxy for socio-economic differences in Brazilian children [19]. If skin color had not been observed one could argue that the difference between test and control groups could be related to higher number of white participants in one of the groups. The results showed that the majority of the study population was mixed. Two issues have emerged from this figure. The disparity between the estimates for 1,000 children could not be attributed to this variable and the context marked by a low-income area was reinforced.

It may be argued that gender-risk differences associated to the caries experience at baseline on deciduous dentition might express characteristics connected to parental homecare and sugar intake not controlled in the study. However if this had been important the caries incidence density values would have been more favorable for the girls compared to the boys (Table 2).

The outcome was measured in each emerged molar. Then three exposed surfaces at risk per tooth were considered and the number of exposed surfaces per child varied from three to twelve. This assumption supported the estimates of sample size and test power. In line with the pragmatic design of the study, the primary analysis was carried out for the whole study population, whether or not they underwent modified programme, and estimates of outcome were compared between control and test groups. It may be argued that the outcome might not be considered as an independent observation because the consequence in a surface could keep dependence with other tooth surfaces of the individual and this might underestimate the amplitude of the confidence intervals. For controlling this, one variable linked to the individual risk was added to the Poisson regression modeling using the random effects. Generalized estimating equations are used for accounting individual- and cluster-level variation and controlling the precision of the estimates. In addition, an estimate related to the number of surfaces in the timing interval, subject to risk, was thus obtained [23]. The use of the incidence density permitted to include the results of interim examinations which would be lost if had been adopted the caries increment as an effect measure [26]. With these procedures, misleading evidence by problems related to treatment-baseline interactions, statistical power and unit of randomization was avoided [27].

Moreover a sole examiner of high consistency and blinded to the specific programs among the preschools was employed. Initial and advanced non-cavitated carious lesions or those in which cavitation was up to 0.5 mm were not considered as decayed surfaces avoiding difficulties related to the reversal phenomenon of the lesions. For this reason it was assumed that caries was underscored in both groups.

Based on the abovementioned aspects, the findings can be considered valid and can be ascribed to the professional cross-brushing adopted in the modified program. Reasons of ethical order did not recommend the assigning of a control group without exposure to fluoride dentifrice, a preventive measure of acknowledged evidence [28,29]. The observed effect can be related to the lower timing interval and to the technique. Both aspects are related to the modification of conditions for plaque accumulation on molar surfaces [5]. Moreover such alterations could facilitate the action of fluoride on the occlusal surface, an area less susceptible to fluoride treatments [3]. In addition, the lower timing interval requires more human and material resources. For this reason, beyond the effectiveness investigated in this study it is important to assess the marginal costs related to the number of avoided lesions. This subject matter will be dealt in other publication.

Although the difference has been no statistically significant for the population as a whole, the data can be relevant from a scientific point of view. The absence of evidence of benefit for the outcome should not be interpreted as evidence of absence of effect but as benefit directly associated with risk conditions of the individuals. To find differences in short-term studies where both control and test groups are subject to regimes of similar exposure to fluoride is more difficult than between groups of distinct regimes. Based on the short term of the follow-up, the exposure of both groups to fluoridated water and dentifrice as well as the significant outputs obtained among the boys, it is licit to project a relevant effect in a larger time period in those more vulnerable individuals of the population. The found results strongly encourage further studies in a non-fluoridated area.

## Conclusion

The modified program of supervised toothbrushing was effective among the boys. The results suggest that the adoption of this practice can increase the effectiveness of such programs in those more vulnerable individuals. Useful information for evaluating and reorienting school-based supervised toothbrushing programs was produced suggesting a dental caries significant decline in a broader population which can represent substantial reduction of dental care needs through training auxiliary personnel operating in dental public services. This study has implications for school-based supervised toothbrushing programs directed to children from 5 to 8 years-old. Evidence related to professional cross-brushing on erupting permanent molars within a community intervention as presented in this paper encourages decision-makers for adopting feasible measures to include this practice on such programs.

## Competing interests

The author declares that they have no competing interests.

## Authors' contributions

PF conceived the research question, designed the study and elaborated the protocol. PF also analyzed the data and wrote the manuscript. He also read and approved the final manuscript.

## Pre-publication history

The pre-publication history for this paper can be accessed here:

http://www.biomedcentral.com/1472-6831/11/11/prepub
